# Cranial geometry in patients with dystonia and Parkinson’s disease

**DOI:** 10.1038/s41598-023-37833-3

**Published:** 2023-07-07

**Authors:** Joji Fujikawa, Ryoma Morigaki, Kazuhisa Miyake, Taku Matsuda, Hiroshi Koyama, Teruo Oda, Nobuaki Yamamoto, Yuishin Izumi, Hideo Mure, Satoshi Goto, Yasushi Takagi

**Affiliations:** 1 Department of Advanced Brain Research, Institute of Biomedical Sciences, Graduate School of Medicine, Tokushima University, 3-18-15 Kuramoto-Cho, Tokushima, Tokushima 770-8503 Japan; 2 Department of Neurosurgery, Institute of Biomedical Sciences, Graduate School of Medicine, Tokushima University, 3-18-15 Kuramoto-Cho, Tokushima, Tokushima 770-8503 Japan; 3 Department of Neurology, Institute of Biomedical Sciences, Graduate School of Medicine, Tokushima University, 3-18-15 Kuramoto-Cho, Tokushima, Tokushima 770-8503 Japan; 4 Parkinson’s Disease and Dystonia Research Center, Tokushima University Hospital, 2-50-1 Kuramoto-Cho, Tokushima, Tokushima 770-8503 Japan; 5 Center for Neuromodulation, Kurashiki Heisei Hospital, 4-3-38 Oimatsu-Cho, Kurashiki, Okayama 710-0826 Japan; 6 Center for Drug Discovery and Development Sciences, Research Organization of Science and Technology, Ritsumeikan University, 56-1 Toujiinkita-Machi, Kita-Ku, Kyoto, Kyoto 603-8577 Japan

**Keywords:** Dystonia, Neurodegenerative diseases, Parkinson's disease, Anatomy, Musculoskeletal system, Bone, Skeleton

## Abstract

Abnormal skull shape has been reported in brain disorders. However, no studies have investigated cranial geometry in neurodegenerative disorders. This study aimed to evaluate the cranial geometry of patients with dystonia or Parkinson's disease (PD). Cranial computed tomography images of 36 patients each with idiopathic dystonia (IDYS), PD, and chronic subdural hematoma (CSDH) were analyzed. Those with IDYS had a significantly higher occipital index (OI) than those with CSDH (*p* = 0.014). When cephalic index (CI) was divided into the normal and abnormal groups, there was a significant difference between those with IDYS and CSDH (*p* = 0.000, α = 0.017) and between PD and CSDH (*p* = 0.031, α = 0.033). The age of onset was significantly correlated with the CI of IDYS (τ = − 0.282, *p* = 0.016). The Burke–Fahn–Marsden Dystonia Rating Scale motor score (BFMDRS-M) showed a significant correlation with OI in IDYS (τ = 0.372, *p* = 0.002). The cranial geometry of patients with IDYS was significantly different from that of patients with CSDH. There was a significant correlation between age of onset and CI, as well as between BFMDRS-M and OI, suggesting that short heads in the growth phase and skull balance might be related to the genesis of dystonia and its effect on motor symptoms.

## Introduction

Dystonia is a neurological disorder characterized by involuntary movements and abnormal postures due to persistent or intermittent muscle contractions^1^. Parkinson's disease (PD) is the second most common neurodegenerative disease and is caused by the progressive loss of dopaminergic neurons in the midbrain^2^. Its cardinal motor symptoms include tremor, rigidity, bradykinesia, akinesia, and postural instability. Both dystonia and PD are representative basal ganglia diseases associated with motor symptoms. The precise etiologies of dystonia and PD remain unknown.

Plagiocephaly is a condition in which an infant's skull is slanting or warped. In severe cases, plagiocephaly and a short head in infants may increase the risk of low developmental scores^3,4^, and auditory processing disorders^5^. These conditions generally emerge during the infant's developmental stages, when the head is kept in the same position, for instance, due to maintaining a continuous supine position. Studies evaluating skull shape, such as plagiocephaly, have been conducted primarily on infants, and, recently, helmet therapy to treat plagiocephaly has become a popular approach.

Parameters commonly used to evaluate plagiocephaly include the cranial vault asymmetry index (CVAI), which uses the skull diagonal to evaluate asymmetry, and the cephalic index (CI), which evaluates the shape of the head. However, few studies have focused on skull geometry in adults. In a cadaver study, 19 of 65 specimens (29%) showed irregular undulating and thickening of the frontal bone internal surface, of which 5 specimens (26%) were of patients diagnosed with neurological disorders, including Alzheimer's disease, dementia, depression, and PD^6^. Nagaishi et al. used computed tomography (CT) to investigate skull morphological changes^7^ and showed that cranial shape significantly differed between patients with moyamoya disease, cervical internal carotid artery stenosis, depression, or schizophrenia and control participants. Thus, abnormal skull shape may be related to brain dysfunction and disease. To the best of our knowledge, no studies have investigated this possibility in neurodegenerative disorders such as dystonia and PD. Deep brain stimulation (DBS) is often used for treating dystonia and PD^8^. Understanding the cranial geometry is important when performing DBS surgery, where electrodes are precisely inserted at microscopic targets in the deep brain such as the subthalamic nucleus and ventral intermediate nucleus. This study aims to evaluate the cranial geometry of patients with dystonia and PD.

## Materials and methods

### Study population

Clinical and CT findings from 108 Japanese patients treated at Tokushima University Hospital between 2004 and 2022 were retrospectively reviewed and analyzed for skull shape and size. The power analysis showed that with a sample size of 108 patients, assumed effect size (f) of 0.3, and α error of 0.05, the actual power of this clinical trial reached 0.80 in the chi-square test, and the sample size was determined on this basis. Among the included patients, 36 had idiopathic dystonia (IDYS), 36 had PD, and 36 had chronic subdural hematoma (CSDH). Consecutive patients aged at least 20 years were enrolled. Patients with hereditary PD were excluded. All patients with IDYS or PD had undergone DBS surgery and had a confirmed diagnosis. Owing to the difficulty of collecting CT images from sufficient healthy individuals, patients with CSDH were categorized as controls. Patients with CSDH were also selected if they had no history of neurodegenerative disease. The average age of the participants was 47.3 years (median 46.5 years) for IDYS, 63.9 years (median 66.0 years) for PD, and 77.6 years (median 80.5 years) for CSDH. The participants included 27 men and 9 women with IDYS, 22 men and 14 women with PD, and 26 men and 10 women with CSDH. In patients with PD, to adjust for sex differences, all male patients encountered during the study period were enrolled and female patients were added. All data were obtained from the medical records of Tokushima University Hospital.

### Cranial vault asymmetry and cephalic and occipital indices

**Figure 1:**
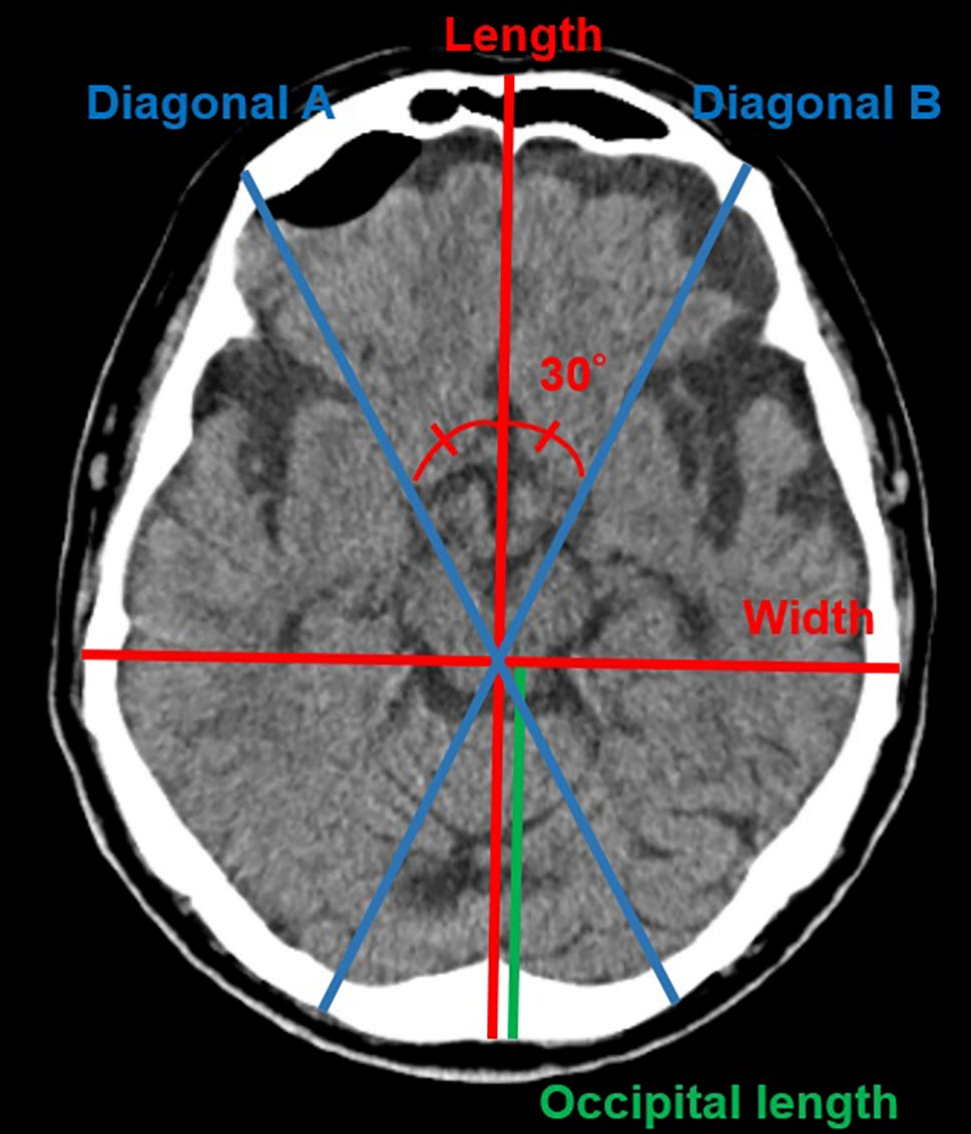
Cranial computed tomography image showing representative lines used to calculate the cephalic index, cranial vault asymmetry index, and occipital index.

The helical CT scan was performed with an axial slice thickness of 1 mm. Slices for analysis were created using iNtuition Viewer V4.4. 12.100 (TeraRecon, San Mateo, CA, USA). The measurement and parameter calculation methods were based on previous studies^7,9^. After defining a horizontal plane from a line through the middle of the eye and ear canal, orbital slices parallel to this plane were created by multiplanar reconstruction. From these slices, a slice at the level of the superior orbital rim was selected for measurement and analysis. CI, CVAI, and occipital index (OI) were used as analysis items (Fig. 1). CI is the percentage of head width to head length (Eq. 1), and length is the distance of the most prominent part.

1 $$Cephalic\,index\,\left(CI\right)=\frac{width}{length}\times 100$$

The CVAI was calculated by drawing two 30° diagonals and dividing the difference between the long and short diagonals by the short diagonal (Eq. 2).

2 $$Cranial\,vault\,asymmetry\,index\,\left(CVAI\right)=\frac{|diagonal\,A-diagonal\,B|}{short\,diagonal}\times 100$$

OI was calculated by splitting the head length into frontal and occipital lengths based on the position of the head width.

3 $$Occipital\,index\,\left(OI\right)=\frac{occipital\,length}{length}\times 100$$

Figure 1 illustrates the lines used to calculate CI, CVAI, and OI. Analysis items were calculated using MATLAB software version R2020b (The MathWorks, Inc. Natick, MA, USA). Here, the normal ranges for CI and CVAI were 76–80.9%^10^ and < 3.5^11^, respectively, and all other values were considered abnormal. In addition, age at onset of IDYS and PD and Burke–Fahn–Marsden Dystonia Rating Scale (BFMDRS) scores for IDYS were obtained to examine the relationship between disease characteristics. For the BFMDRS score, the motor scale (BFMDRS-M) and disability scale (BFMDRS-D) were used.

### Statistical analysis

Differences in the age and sex distributions of the participants were assessed using analysis of variance (ANOVA) and the Kruskal–Wallis rank-sum test, respectively. Differences between groups using CVAI, CI, and OI measures were assessed using the Kruskal–Wallis rank-sum test or ANOVA for non-normally distributed or normally distributed data, respectively, followed by post-hoc analyses using the Wilcoxon rank sum exact test. Cliff's delta was used to obtain the effect size. In addition, CVAI and CI were divided into two groups (normal or abnormal values), and group differences were evaluated using the chi-square test. Cramer's *V* was used to measure the strength of relationship between each group. Multiple comparisons were made using Ryan's method. The correlations among CVAI, CI, OI, age of onset, and BFMDRS score were evaluated using Kendall's rank correlation τ and Pearson's product-moment correlation. Differences were considered statistically significant at *p* < 0.05, except for those obtained using Ryan’s method. For comparisons of three groups, significant *p* values were corrected using Bonferroni's correction. Statistical analyses were performed using R version 4.2.1^12^.

### Ethical approval

The study was approved by the Ethics Committee of Tokushima University Hospital (approval number 3743-2, date of approval Dec 19, 2022) and was performed in accordance with the principles detailed in the Declaration of Helsinki. The requirement for written informed consent was waived by the Ethics Committee due to the retrospective design of the study. All procedures were carried out in accordance with the relevant guidelines and regulations.

## Results

**Figure 2:**
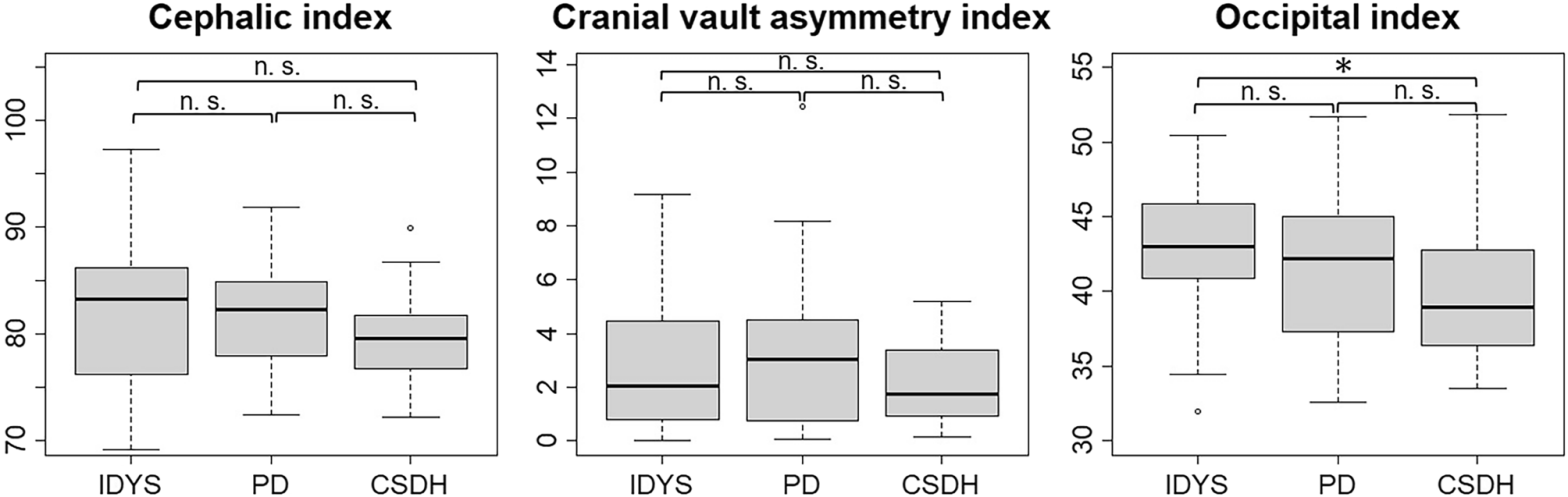
Box plot graphs showing the cephalic index, cranial vault asymmetry index, and occipital index measurements for the idiopathic dystonia (IDYS), Parkinson's disease (PD), and chronic subdural hematoma (CSDH) groups. The IDYS group had a significantly higher occipital index than the CSDH group (*p* = 0.014). *Significantly different after Bonferroni correction (*p* < 0.02). *n. s*. not significantly different.

First, we examined whether there were any significant differences in the age and sex distributions among the groups. The three groups significantly differed in age (*p* = 0.000); however, since a morphological plateau is reached at approximately 20 years of age, this did not pose any issues for the analysis. There were no significant differences in sex among the groups (*p* = 0.403). We performed the analysis in three counties with IDYS, PD, and CSDH. The CI, CVAI, and OI measurements for each group are shown in Fig. 2. The Kruskal–Wallis rank-sum test or ANOVA was used to examine differences among the three groups for each measure (CVAI, CI, and OI). The Kruskal–Wallis rank-sum test for CVAI and OI revealed *p* values of 0.427 and 0.032, respectively. The ANOVA for CI revealed an *f* value of 1.577 and a *p* value of 0.211. The difference was significant only for OI. Post-hoc analysis revealed a significant difference in OI between the IDYS and CSDH groups (*p* = 0.014, δ = 0.336). The effect size by Cliff's delta was medium. These measurements of OI (mean ± SD) were 42.481 ± 4.355 for the IDYS group and 39.955 ± 4.797 for the CSDH group. No statistically significant differences were observed in the other parameters.

We divided the patients into the normal and abnormal groups for CI and CVAI, for which normal values were defined (CI 76–80.9%^10^ CVAI: < 3.5^11^) and evaluated the differences between the groups using the chi-square test. The percentage of abnormal CI values was 86.1%, 72.2%, and 47.2% for the IDYS, PD, and CSDH groups, respectively. For CVAI, the percentages were 36.1%, 44.4%, and 22.2% for the IDYS, PD, and CSDH groups, respectively. Results from the chi-square test showed significantly different CI (χ^2^(2) = 12.963, *p* = 0.002, *V* = 0.346). The strength of relationship by Cramer’s *V* was medium. Multiple comparisons using Ryan’s method showed significantly different CI between the IDYS and CSDH groups (*p* = 0.000, α = 0.017) and between the PD and CSDH groups (*p* = 0.031, α = 0.033). However, there was no significant difference in CVAI between the groups (χ^2^(2) = 4.029, *p* = 0.133).

Next, we evaluated correlations between CVAI, CI, and OI and age of onset in patients with IDYS and PD and the BFMDRS score in patients with IDYS using Kendall's rank correlation τ or Pearson's product–moment correlation. The age of onset was 33.0 years (median = 35.5 years) for IDYS and 53.3 years (median 53.0 years) for PD. Age of onset was only correlated with CI in the IDYS group (τ = − 0.282, *p* = 0.0164). In the IDYS group, the BFMDRS-M score was 26.6 (median 22.3) and BFMDRS-D score was 9.82 (median 6). The BFMDRS-M score showed a significant correlation with OI in patients with IDYS (τ = 0.372, *p* = 0.002). No correlations were found for any of the items in the BFMDRS-D.

## Discussion/conclusion

Reportedly, 95% of the adult brain volume is achieved at age 9^13^, with a morphological plateau at approximately 20 years of age. Bastir et al. showed that the skull and intracranial spaces reach their mature size and shape by 15.7 years of age, although different parts of the cranium mature at different ages^14^. Once the shape is stabilized, the size and shape usually do not change. Therefore, CT is a reliable method for evaluating the cranial shape. This study is the first to investigate the cranial geometry of patients with dystonia or PD, and the indices used to assess cranial shape were CVAI, CI, and OI. CVAI can quantify cranial asymmetry and has been proposed for the diagnosis and classification of plagiocephaly. Within this study, CVAI values did not differ significantly between the groups. However, a higher percentage of abnormalities were found in the IDYS and PD groups than in the CSDH group (IDYS: 36%, PD: 44%, CSDH: 22%).

CI is an index used to assess short heads in infants. In the CSDH group, the abnormal CI rate was approximately 47%, while it was 86% in the IDYS group and 72% in the PD group, indicating a high percentage of abnormalities in patients with IDYS or PD. It has been reported that Japanese infants tend to have shorter heads than Caucasian children because they are traditionally kept on their backs^15^. When divided into the abnormal and normal groups, statistically significant differences were observed between the IDYS and CSDH groups and between the PD and CSDH groups, suggesting that IDYS and PD tend to affect the shape of the head may be related to the disease. Interestingly, CI was negatively correlated with age of onset in the IDYS group, suggesting that short heads in the growth phase might be correlated with the pathophysiology of dystonia. The CI value did not differ between the PD group and the other groups, although the percentage of abnormalities was higher. High CI has been reported in patients with schizophrenia^7^. A cadaver study identified irregular undulating and thickening of the frontal bone internal surface in patients with Alzheimer’s disease, dementia, depression, and PD^6^. These findings suggest a potential association between cranial morphology and neuropsychiatric disorders, including PD.

OI is a measure of the length of the posterior relative to the entire cranium^7^. During infancy, the back of the head is flattened when the infant is maintained in a supine position continuously, and this translates to a low OI value (flat head). In the present study, significant differences were observed between the CSDH and IDYS group. The skull balance differed between the IDYS group and the other groups, showing a greater proportion of the occipital area. Furthermore, OI was positively correlated with BFMDRS-M score in the IDYS group, indicating that a higher OI correlates with worse motor symptoms. As an example of OI being associated with disease, Nagaishi et al. reported a high OI in cervical internal carotid artery stenosis and depression, suggesting that differences in head balance, such as OI, may influence disease^7^.

There are two limitations of this study. First, only planar analysis was performed; a three-dimensional survey could capture more detailed characteristics. Second, we used CSDH patients as controls; however, the possibility remains that they differ from healthy controls. Although CSDH is not a neurodegenerative disease, the inclusion of healthy participants as controls would have been ideal to minimize unexpected bias.

In conclusion, the cranial geometry of patients with IDYS was significantly different from that of patients with CSDH. There was a significant correlation between age of onset and CI, as well as between BFMDRS-M score and OI, suggesting that short heads in the growth phase and skull balance might be related to the development of dystonia and motor symptoms. However, our results only show a correlation, and further investigation is needed to determine a direct causal relationship. Cranial geometry might be one factor in delineating the pathogenesis of IDYS and PD.

## Data Availability

The datasets generated and/or analyzed during the current study are available from the corresponding author upon reasonable request.
